# Effectiveness of a smartphone app (Drink Less) versus usual digital care for reducing alcohol consumption among increasing-and-higher-risk adult drinkers in the UK: a two-arm, parallel-group, double-blind, randomised controlled trial

**DOI:** 10.1016/j.eclinm.2024.102534

**Published:** 2024-03-24

**Authors:** Melissa Oldham, Emma Beard, Gemma Loebenberg, Larisa Dinu, Colin Angus, Robyn Burton, Matt Field, Felix Greaves, Matthew Hickman, Eileen Kaner, Susan Michie, Marcus Munafò, Elena Pizzo, Jamie Brown, Claire Garnett

**Affiliations:** a Department of Behavioural Science and Health, University College London, UK; b School of Health and Related Research, University of Sheffield, Sheffield, UK; c Office for Health Improvement and Disparities, London, UK; d Institute of Psychiatry, Psychology and Neuroscience, Kings College London, UK; e Department of Psychology, University of Sheffield, Sheffield, UK; f Department of Primary Care and Public Health, Imperial College London, London, UK; g NICE (National Institute for Health and Care Excellence), London, UK; h Population Health Sciences, Bristol Medical School, University of Bristol, Bristol, UK; i Population Health Sciences Institute, Newcastle University, Newcastle upon Tyne, UK; j Centre for Behaviour Change, University College London, London, UK; k School of Psychological Science, University of Bristol, Bristol, UK; l MRC Integrative Epidemiology Unit, University of Bristol, Bristol, UK; m Department of Applied Health Research, University College London, London, UK

**Keywords:** Alcohol reduction app, MHealth, Randomised controlled trial, Drink less, Digital intervention

## Abstract

**Background:**

Digital interventions, including apps and websites, can be effective for reducing alcohol consumption. However, many are not evidence- or theory-informed and have not been evaluated. We tested the effectiveness of the Drink Less app for reducing alcohol consumption compared with usual digital care in the UK.

**Methods:**

In this two-arm, parallel group, double-blind, randomised controlled trial, we enrolled increasing-and-higher-risk drinkers (AUDIT ≥ 8) in the UK, who were motivated to reduce their alcohol consumption and willing to use a digital intervention to do so, via online methods. Participants were randomly assigned (1:1), using an online algorithm, to receive a web link to download the Drink Less app (intervention) or to the NHS alcohol advice webpage (usual digital care). Researchers were masked to group allocation. Participants were followed up at one, three and six months. The primary outcome was self-reported weekly alcohol consumption at six months, adjusting for baseline consumption. The full analytic sample was used in most analyses, though missing data was treated in different ways. The primary, pre-registered intention-to-treat analysis assumed baseline-carried-forwards. Secondary pre-registered analyses also focused on the full analytic sample and used alternatives including multiple imputation and last observation carried forwards. This trial is registered with the ISRCTN registry, ISRCTN64052601.

**Findings:**

Between 07/13/2020 and 03/29/2022, 5602 people were randomly assigned to the Drink Less app (n = 2788) or comparator (n = 2814) groups. Six-month follow-up rates were 79% and 80%, respectively. The primary pre-registered conservative intention-to-treat approach assuming non-responders were drinking at baseline levels of consumption, found a non-significant greater reduction of 0.98 units in weekly alcohol consumption in the intervention group at 6-month follow-up (95% CI −2.67 to 0.70). The data were insensitive to detect the hypothesised effect (Bayes factor = 1.17). Data were not missing completely at random, with 6-month follow-up rates differing in terms of education, occupation, and income. We therefore conducted the pre-registered sensitivity analysis using multiple imputation, showing that the *Drink Less* app resulted in a 2.00-unit greater weekly reduction at 6-month follow-up compared with the NHS alcohol advice webpage (95% CI −3.76 to −0.24). Fewer than 0.1% of participants in both arms who responded to one, three or six-month follow-up reported adverse events linked to participation in the trial.

**Interpretation:**

The *Drink Less* app may be effective in reducing the alcohol consumption in increasing-and-higher-risk drinkers motivated to reduce their consumption.

**Funding:**

10.13039/501100001921NIHR Public Health Research Programme.

**Research in context**

**Evidence before this study**

We searched PubMed using the search terms “alcohol reduction app” and “UK general population” from database inception up to 15 02, 2024. Of the four publications, three were focused on the Drink Less app. The fourth was focused on an evaluation of the Drinks:Ration app, which aims to reduce alcohol harms amongst UK veterans. Additional articles were identified through stratified searches on PubMed.

Digital interventions have the potential to reach large numbers of increasing-and-higher-risk drinkers at relatively low costs and overcome barriers of face-to-face brief interventions. There have been promising evaluations of apps in Sweden and Australia, where use of apps seemed to lead to alcohol reduction. However, despite the availability of hundreds of alcohol-related apps in commercial app stores, none have been evaluated in a randomised controlled trial (RCT) among a general population of adults in the UK.

**Added value of this study**

This study is the first large RCT of 5602 increasing-and-higher-risk drinkers in the UK, testing the effectiveness of an app, Drink Less, for alcohol reduction relative to usual digital care.

The Drink Less app showed a non-significant benefit relative to usual digital care in the primary analysis which assumed no change from baseline for those who did not respond at six-month follow-up. A prespecified sensitivity analysis using multiple imputation for missing data indicated a significant difference between the two groups.

**Implications of all the available evidence**

Drink Less may offer an effective, individual-level intervention that is scalable and could reach a large proportion of the UK population at a low incremental cost. This is particularly important given that following the COVID-19 pandemic, public health infrastructure has been restructured and the UK has announced a new strategic focus on digital public health with key services pivoting largely to remote delivery.

## Introduction

There is a dose response relationship between alcohol consumption and alcohol-related harms.^1^ Fewer than 7% of increasing-and-higher-risk drinkers (Alcohol Use Disorder Identification Test: AUDIT score ≥8) receive face-to-face interventions in primary care to support alcohol reduction.^2^ Barriers to the delivery of these interventions by practitioners are time and low confidence about discussing alcohol with patients.^3^ Digital interventions can be effective for reducing alcohol consumption,^4^ and avoid many of the barriers of face-to-face delivery with low incremental costs.^5^ However, most digital alcohol interventions that have been evaluated are web-based and there has been little evaluation of the effectiveness of apps in the UK.^4^ The aim of this randomised controlled trial (RCT) was to evaluate the effectiveness of the recommendation of the evidence- and theory-informed app, Drink Less, in reducing alcohol consumption among increasing-and-higher-risk drinkers in the UK, compared with the recommendation of usual digital care.

A 2017 Cochrane review found digital interventions may reduce alcohol consumption, with an average reduction of 23 g of alcohol (2.9 UK units) per week compared with usual digital care.^4^ However, only one of the digital interventions used a smartphone app, and this RCT was conducted in Sweden amongst university students.^6^ Since the 2017 review, a number of alcohol reduction apps have been evaluated. One app resulted in a reduction in standard drinks and heavy drinking days among a student sample in Switzerland after one year.^7^ In Australia, the Daybreak app resulted in reductions in alcohol consumption relative to baseline amongst users,^8^ though due to technical errors a proportion of the control group in this study accessed the intervention.

Around 84% of people in the UK have access to a smartphone and coverage is ≥ 95% among those aged 18–54.^9^ As such, smartphone apps are a promising mode of digital intervention delivery in the UK. Smartphone apps also have other advantages over websites, including the ability to be used without internet access and to provide a more tailored user-experience. In the UK, evaluations of alcohol reduction apps have been limited. An app designed for reducing alcohol use amongst veterans resulted in a reduction in AUDIT scores after 84 days but this effect was not present at the last follow-up.^10^ In an observational study among the general population, engaged users of the Drinkaware app appeared to reduce their alcohol consumption over time,^11^ but this study did not have a comparator group so reductions in alcohol use cannot be attributed to the app. Drinkaware is funded by voluntary and unrestricted donations from major UK alcohol producers and related industries. Despite the availability of hundreds of alcohol-related apps in commercial app stores, none have been evaluated in an RCT among the general population of adults in the UK.^4^ Furthermore, many of the smartphone apps available in the app store have been developed without reference to scientific evidence or theory.^12^

The Drink Less app was designed to help increasing-and-higher-risk drinkers reduce their alcohol consumption, and was developed and refined using a systematic and iterative process.^13^^,^^14^ The development of the Drink Less app was informed by multiple sources of evidence^15^^,^^16^ and has been described in detail elsewhere.^13^ The development and evaluation of Drink Less was also guided by the UK Medical Research Council's guidance on complex interventions^17^ and the Multiphase Optimisation Strategy.^18^ A factorial screening trial showed that combinations of the intervention components were effective in reducing alcohol consumption.^14^ Interventions developed by researchers are not always made available to the wider public because of a lack of evidence or resource constraints. The Drink Less app is engaging^14^ and already widely used (>70,000 unique users since its launch in 2016), highly rated by users (4.5/5 stars as of May 2023) and highly visible (included in the top 5 results for ‘alcohol’ searches) on the Apple app store.^19^ The Drink Less app offers a scalable population-level intervention.

This study aimed to evaluate the effectiveness of recommending use of the Drink Less app, compared with recommending the use of usual digital care in an RCT (iDEAS trial; iOS Drink Less, evaluating the Effectiveness of an Alcohol Smartphone app).

This study addresses the following research questions:

Among increasing-and-higher-risk drinkers, does the digital recommendation to use Drink Less compared with the NHS alcohol advice webpage reduce weekly alcohol consumption (in UK standard units) at 6-month follow-up, adjusting for baseline consumption?

Among increasing-and-higher-risk drinkers, does the digital recommendation to use Drink Less compared with the NHS alcohol advice webpage:

Reduce weekly alcohol consumption (in UK standard units) at 1- and 3-month follow-up, adjusting for baseline consumption?

Reduce heavy episodic alcohol consumption at 6-month follow-up?

Reduce full adapted AUDIT score at 6-month follow-up?

Reduce alcohol-related problems and injury, and use of healthcare services at 6-month follow-up?

Improve health-related quality of life at 6-month follow-up?

Result in differential changes for lighter versus heavier drinkers (based on their consumption at baseline)?

## Methods

### Study design

A two-arm, parallel group, double-blind, randomised controlled trial (RCT) was conducted remotely (online, via phone call or post) with a 1:1 allocation comparing the recommendation of the intervention (Drink Less) with usual digital care (the NHS alcohol advice webpage). There was an embedded process evaluation involving: i) quantitative analysis of engagement with the intervention which demonstrated that 78% of participants downloaded the Drink Less app and self-reported adherence mediated alcohol reduction at 6-months,^20^ and ii) qualitative analysis of intervention acceptability which demonstrated that the Drink Less app was considered to be an acceptable tool.^21^ The study was conducted remotely with participants who lived in the UK. Ethical approval was obtained from UCL Research Ethics Committee [16,799/001]. The trial was registered (ISRCTN64052601), the data analysis plan was pre-registered^22^ and updates logged with the NIHR^23^ before data were unblinded.

### Participants

Participants were included if they: were aged ≥18, lived in the UK, were increasing-and-higher-risk drinkers (AUDIT score ≥ 8), had access to an iOS device (i.e. iPhone, iPod touch or iPad), and wanted to drink less alcohol. Participants were excluded if they reported being unwilling to complete follow-up assessments at baseline or were unable to read English.

Recruitment ran from July 2020 to March 2022 with the final follow-up collected in October 2022. Participants were recruited via a multi-pronged strategy including: an advertisement on the NHS webpage on alcohol support; social media (e.g. Facebook adverts), radio advertising, press releases, and local advertising through health care providers (through emails and posters in local surgeries). Advertisements were co-developed with public representatives. The cost and effectiveness of different recruitment strategies are reported in further detail in a separate paper.^24^ Participants received a £6 voucher following the completion of the 1- and 3-month follow-up surveys. For the 6-month follow-up survey, participants received a £12 voucher for its completion with an additional £12 voucher if the survey was completed within 24 h.

### Randomisation and masking

Randomisation was generated by an online automated algorithm (at a ratio of 1:1), which tracked counts to ensure each intervention was displayed equally. Allocation was online and participants and researchers were masked to study arm. If participants raised technical queries the researcher would be unblinded, participants seeking technical assistance received no information on the intervention in the other condition and so were not unblinded. The trial statistician had no contact with participants throughout the trial and remained blinded for the analysis. At the end of the baseline survey, participants were randomised to view one of two pages with the recommendation to either download Drink Less (intervention) or the recommendation to view the NHS alcohol advice webpage (comparator). This was a pragmatic trial testing the recommendation of the app versus recommendation of the alcohol advice webpage. Participants did not have to use the tool to be eligible for follow-up and there were no incentives for using or engagement with the tool. Participants scoring 20 or more on the baseline AUDIT were advised that they may be at risk of alcohol dependence and that they should contact a healthcare professional, though were still able to take part in the trial.

Drink Less consists of eight evidence-based modules to help users change their drinking behaviour: Goal Setting, Self-Monitoring and Feedback, Action Planning, Normative Feedback, Cognitive Bias Re-Training, Insights, Behavioural Substitution, and Information about Antecedents. See Supplementary Table S1 for more information including the details of each module, the key features and included behaviour change techniques.

The webpage contains tips for cutting down on alcohol consumption such as planning, setting a budget and switching to smaller or weaker strength drinks. This is presented alongside a number of benefits for cutting down for physical and mental health including; weight loss, and improvements in mood and sleep.^25^ See Supplementary Table S2 for a summary of the behaviour change techniques present on the NHS alcohol advice webpage.

### Procedures

Participants self-enrolled into the study and responded to a web-based screening questionnaire, which assessed the inclusion and exclusion criteria, including the full AUDIT. Those eligible were asked to provide online informed consent and complete sociodemographic measures and contact details (email address, telephone number and postal address) for follow-up assessments. Participants were then randomised and provided with the recommendation to either download the Drink Less app (intervention) or view the NHS alcohol advice webpage (comparator).

Follow-up assessments were conducted one, three and six months after baseline. The 6-month follow-up survey assessed primary and secondary outcome measures. The 1- and 3-month follow-up surveys only assessed the primary outcome measure. We attempted to recontact participants for 30 days from their first invitation to complete the survey at 1- and 3-months. To maximise data retention and to allow time for postal responses to be returned at 6-month follow-up, data provided up to two weeks after this final contact were accepted (Appendix 2 for detailed follow-up procedure).

Participants were asked whether they were happy to take part in a follow-up interview and whether they had used any other forms of support for alcohol reduction at the 6-month follow-up. The schedule of enrolment and follow-up assessment for trial participants is described in more detail in the published protocol.^22^^,^^23^

On completion of the trial participants were given a list of further support including both the Drink Less app and the NHS alcohol advice webpage.

### Outcomes

The primary outcome measure was self-reported weekly alcohol consumption estimated over the last month, in UK standard units, at 6-month follow-up adjusted for baseline weekly alcohol consumption. Weekly alcohol consumption was derived from the extended quantity-frequency questions of the AUDIT, adjusting for heavy episodic use (question 3 of the AUDIT; see Appendix 3 for details).

An error was made on questions 1 and 2 of the AUDIT questionnaire, see Appendix 3 for details. Due to this error, extended responses were not collected until the 01/15/2021 for participants selecting ‘10 or more units’ to question 2 of the AUDIT, these data were imputed, following recommendations 20 imputed datasets were used and combined using Rubin's rules.^26^ This equates to 656 participants (12% of the total expected) at baseline, 186 (3%) at 1-month, 79 (1%) at 3-month and 1 (<0.1%) at 6-month follow-up.

The secondary outcomes were:

Weekly alcohol consumption at 1- and 3-month follow-up adjusted for baseline weekly alcohol consumption.

Heavy episodic alcohol use (measured using AUDIT question 3^27^) at 6-month follow-up.

Full adapted AUDIT score^27^ at 6-month follow-up.

Alcohol-related problems or consequences and alcohol-related injury (measured using the Alcohol Short Index of Problems^28^) at 6-month follow-up.

Use of healthcare services (measured using the Service Use Questionnaire^29^) at 6-month follow-up.

Health-related quality of life (measured using the EQ-5D-5L^30^) at 6-month follow-up.

Change over time in self-reported weekly alcohol consumption at 1-, 3- and 6-months.

Interactions between group allocation and baseline drinking on the primary outcome (alcohol consumption at six months) and on two secondary outcomes (self-reported alcohol consumption at one and three months).

Participants were asked to report any unexpected consequences, adverse events or other harms from participating in the study in an open-ended question at 1-, 3- and 6-month follow-up. Free text responses underwent content analysis and were categorised as: 1) a medical problem related to trial participation or alcohol reduction, 2) a medical problem with an unclear link to participation or reduction, 3) a medical problem unrelated to participation or reduction, or 4) not a medical problem. See Appendix 4 for more detail.

Sociodemographic measures were assessed at baseline: age (in years, continuous), gender (‘male’, ‘female’, ‘other’, ‘prefer not to say’), ethnicity (% white), education (% post-16 educational qualifications), occupation [to derive social grade AB, C1, C2, D, E dichotomised into: ABC1 (managerial, professional and intermediate occupations) versus C2DE (skilled, semi-skilled, unskilled manual and lowest-grade worked or unemployed)], and annual household income (% >£26,000). Education, occupation and income are indicators of socioeconomic status which has been shown to impact on the efficacy of digital interventions.^31^^,^^32^

Recruitment took place during the COVID-19 pandemic and associated lockdowns in the UK, which had a polarising effect on drinking, particularly among heavier drinkers.^33^ Participants were asked whether they felt COVID-19 was affecting their alcohol consumption. Participants responding “yes” were asked follow-up questions assessing the extent to which the pandemic was affecting their concerns about their alcohol consumption, their motivation to cut down and their patterns of consumption. See Appendix 4 for more detail.

### Statistical analysis

We experienced issues with participant deception throughout the trial, this is described in Appendix 2 and in more detail in a separate paper.^34^ Data were cleaned and follow-up files merged using pandas (see Appendix 2 for further details). Data were analysed using R Studio by the trial statistician who was blinded to participants’ group. Code and data are available on OSF (https://osf.io/2j9df/).

**Fig. 1:**
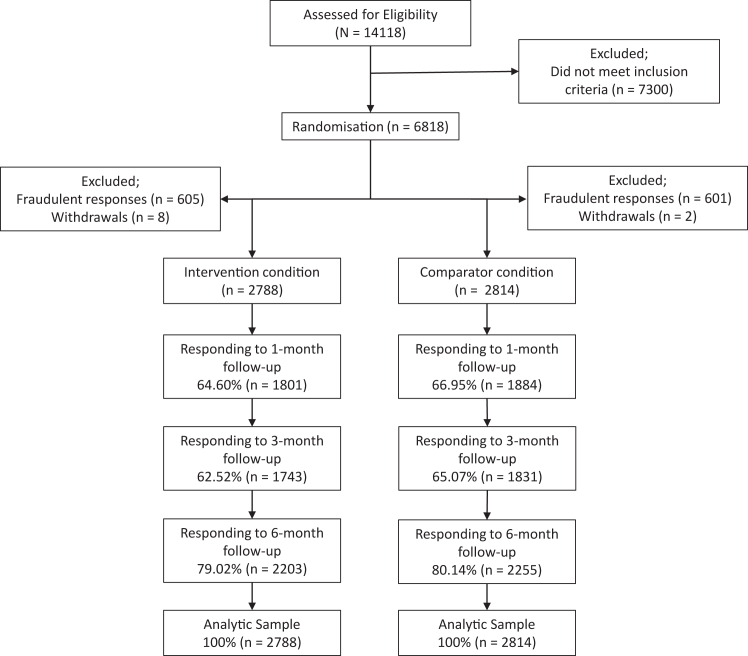
CONSORT diagram of participant flow.

We aimed to recruit a sample size of 5562 participants (2781 in the comparator group and 2781 in the intervention group) with over-recruitment by 50 people to account for possible removals due to duplicate responses or withdrawals, that were detected after data collection closed. This calculation was based on detecting a mean difference reduction of 2 UK units (16 g of alcohol) with an SD of 23 at 90% power with an alpha of 0.05 and a two-tailed test. The estimated effect size is in line with the Cochrane review on digital alcohol interventions^4^ and is roughly equivalent to that found in face-to-face brief intervention outcomes.^35^ The total sample recruited (after duplicate responses and withdrawals were removed) was 5,602, with n = 2814 in the comparator group and n = 2788 in the intervention group. See Fig. 1 for CONSORT diagram.

The trial had an independent Data Monitoring Committee (DMC) who met annually to monitor the data from the trial alongside any ethical or safety reasons why the trial should not continue. The DMC approved updates to the original protocol whilst data collection was ongoing and before unblinding.^23^

The primary analysis used a conservative intention-to-treat approach to missing data with the assumption of no change (6-month follow-up = baseline) for participants who did not respond to follow-up. The effect of group allocation on the primary outcome, weekly alcohol consumption, was examined with a one-way ANCOVA, adjusting for baseline weekly alcohol consumption.

In the event of a non-significant main effect, we planned to calculate a Bayes Factor using a half normal distribution to specify the predicted effect (of a 2 UK unit reduction per week) with a peak at 0 (no effect) and the standard deviation equal to the expected effect size. Bayes Factor is interpreted as the ratio of the likelihood of the observed data occurring under the alternative hypothesis to the likelihood of the observed data occurring under the null hypothesis. We planned to report robustness regions to specify the range of expected effect sizes that support the same conclusion.

Interactions were assessed between group allocation and age, gender, ethnicity, education, occupation, income and COVID-19 question one for primary outcome. Where significant interactions were found the findings were stratified by the variable of interest to explore subgroup effects.

Two sensitivity analyses critically examined the impact of the conservative treatment of missing data on the primary analysis of the primary outcome at six months.

Using multiple imputation for non-responders on baseline characteristics and assuming a normal distribution with a mean of 0 and SD reflecting the variation in change among responders.

Complete case analysis of responders only (i.e. those who completed the 6-month follow-up survey).

The remaining sensitivity analyses used the same approach to missing data (e.g. baseline-carried-forward) as the primary analysis and examined the impact of assumptions about who was included in the final analytic sample (3, 6, 7) or the analysis (4, 5):

Per-protocol approach whereby only participants who reported using the intervention or comparator at 1- or 6-month follow-up are included in the analyses, and participants whose treatment was contaminated are excluded.

Change between baseline and 6-month follow-up in weekly alcohol consumption estimated over the last six months, in standard units, derived from the quantity-frequency questions of the AUDIT.

An instrument variable analysis accounting for non-use in the intervention group and contamination in the comparator by operationalising the difference in app usage between the two groups.

Last observation carried forward.

Counterfactual analysis excluding those who reported not having IOS after randomisation (so could not use the Drink Less app) and first case from duplicate respondents.

Secondary analyses (objectives above) used a combination of ANCOVA, ANOVA, independent t-tests, Kruskal–Wallis tests, Welch's ANOVA, linear and logistic regression, and Chi-squared tests.

We added two unplanned secondary analyses:

All secondary analyses were conducted using multiple imputation for missing data. Data was not missing completely at random and therefore using multiple imputation is the least biased approach to missing data.^36^

To explore the range of effectiveness, we conducted an unplanned sensitivity per-protocol analysis, comparing those using the app in the intervention group to all those in the comparator group. This analysis represented the best-case scenario.

### Role of the funding source

The funder of the study had no role in study design, data collection, data analysis, data interpretation, or writing of the report. MO and CG verified the underlying study data reported in the manuscript, EB conducted the analysis. MO and CG had final responsibility for the decision to submit for publication.

## Results

Fig. 1 shows the flow of participants through the trial. We randomly assigned 5602 people to the Drink Less app (n = 2788) or the comparator (n = 2814). At 6-month follow-up there was a drop-out rate of 20% (n = 1144). Follow-up rates did not differ significantly between groups; 79% (n = 2203) in the intervention group and 80% (n = 2255) in the comparator group (P = 0.32).

The intervention and comparator groups were similar in baseline sociodemographic and drinking characteristics (Table 1). There were no statistically significant between group differences in the COVID-19 measures (Supplementary Table S7). There were differences in characteristics of participants at 6-month follow-up and baseline educational qualifications, occupation, and income (Table 1).

**Table 1:** Participant characteristics overall, stratified by intervention group and by whether they responded at 6-month follow-up.

Variable	Overall (n = 5602)	Comparator group (n = 2814)	Intervention group (n = 2788)	Responded at 6-month follow-up (n = 4458)	No response at 6-month follow-up (n = 1144)	P
Gender %(n)						
Female	57.25 (3207)	66.18 (1581)	58.32 (1626)	56.84 (2534)	58.83 (673)	0.13
Male	42.22 (2365)	43.21 (1216)	41.21 (1149)	42.69 (1903)	40.38 (462)	
Other	0.48 (26)	0.50 (14)	0.43 (12)	0.38 (17)	0.79 (9)	
Prefer not to say	0.07 (4)	0.11 (3)	0.04 (1)	0.09 (4)	0.0 (0)	
Age in years M(SD)	41.64 (12.80)	41.73 (12.92)	41.56 (12.69)	41.82 (13.06)	40.95 (11.76)	0.503
Ethnicity %(n)						
White	94.54 (5296)	94.85 (2669)	94.23 (2627)	94.35 (4206)	95.28 (1207)	0.201
Other	0.37 (21)	0.28 (8)	0.47 (13)	0.40 (18)	0.26 (4)	
Asian	1.71 (96)	1.53 (43)	1.90 (53)	1.86 (83)	1.14 (17)	
Black	0.84 (47)	0.82 (23)	0.86 (24)	0.92 (41)	0.52 (6)	
Chinese	0.16 (9)	0.18 (5)	0.14 (4)	0.20 (9)	0.00 (0)	
Mixed	2.02 (113)	1.99 (56)	2.04 (57)	1.88 (84)	2.53 (31)	
Prefer not to say	0.34 (19)	0.32 (9)	0.36 (10)	0.36 (16)	0.26 (3)	
Not known	0.02 (1)	0.04 (1)	0.0 (0)	0.02 (1)	0 (0)	
Highest educational qualification %(n)						
*A-levels or equivalent*	18.78 (1052)	18.62 (524)	18.94 (528)	18.28 (815)	20.72 (237)	<0.0001
*Bachelors degree of equivalent*	37.52 (2102)	37.85 (1065)	37.20 (1037)	38.60 (1721)	33.30 (381)	
*GCSE/O-level/CSE*	12.73 (713)	12.76 (359)	12.70 (354)	11.46 (511)	17.66 (202)	
*Masters/PhD or equivalent*	18.81 (1054)	18.51 (521)	19.12 (533)	20.03 (893)	14.07 (161)	
*No formal qualifications*	1.45 (81)	1.56 (44)	1.33 (37)	1.28 (57)	2.10 (24)	
*Other*	1.87 (105)	1.92 (54)	1.83 (51)	1.79 (80)	2.19 (25)	
*Still studying*	2.50 (140)	2.70 (76)	2.30 (64)	2.49 (111)	2.53 (29)	
*Vocational qualifications*	6.34 (355)	6.08 (171)	6.60 (184)	6.06 (270)	7.43 (85)	
AUDIT score M(SD)	21.89 (6.66)	21.74 (6.56)	21.86 (6.76)	21.40 (6.70)	23.38 (6.27)	0.503
Occupation %(n)						
*High managerial, administrative or professional*	22.85 (1280)	22.35 (629)	23.35 (651)	22.03 (982)	26.05 (298)	<0.0001
*Intermediate managerial, administrative or professional*	30.61 (1715)	31.13 (876)	30.09 (839)	31.52 (1405)	27.10 (337)	
*Semi and unskilled manual workers*	4.39 (257)	4.66 (131)	4.52 (126)	4.49 (200)	4.98 (63)	
*Skilled manual workers*	7.00 (392)	6.33 (178)	7.68 (214)	6.01 (268)	10.84 (131)	
*State pensioners*	3.70 (207)	3.77 (106)	3.62 (101)	4.04 (180)	2.36 (35)	
*Supervisory, clerical and junior managerial, administrative*	20.64 (1156)	20.50 (577)	20.77 (579)	21.31 (950)	18.01 (233)	
*Unemployed*	10.62 (595)	11.27 (317)	9.97 (278)	10.61 (473)	10.66 (137)	
Income %(n)						
*£26,000 or more*	74.76 (4188)	73.88 (2079)	75.65 (2109)	75.95 (3386)	70.10 (802)	<0.0001
*Less than £26,000*	25.24 (1414)	26.12 (735)	24.35 (679)	24.04 (1072)	29.10 (342)	

Note: M = mean, SD = standard deviation; P values from independent t-test (where assumptions of normality and homogeneity of variance were tested using histograms and Bartlett's test and were met) and Chi-squared (Fisher's exact test for low expected count) as appropriate; In a deviation from our pre-registered protocol we elected not to test for baseline differences between groups according to CONSORT statement that it is superfluous and can mislead investigators and their readers. P values refer to comparison of baseline characteristics between those who responded and did not respond at 6-month follow-up.

These differences in follow up indicate that data is not Missing Completely at Random (MCAR) and that using multiple imputation is a more appropriate approach to missing data than assuming no change from baseline.^36^

### Primary analysis

Raw weekly consumption at baseline and 6-month follow-up are shown in Table 2. The assumptions underlying analysis of covariance (ANCOVA) were tested using Histograms of the residuals to assess normality, a boxplot to check for outliers, Bartlett's test for homogeneity of variance and homogeneity of slopes by plotting the interaction between group assignment and the covariate. All assumptions were met. The primary analysis, using the conservative no-change-from-baseline approach to missing data, did not detect a statistically significant difference between groups (P = 0.26; Adjusted Mean difference = −0.98, 95% CI = −2.67 to 0.70; Mean comparator group: 47.05; Mean in the intervention group: 46.07). A Bayes Factor of 1.17 indicated this analysis was insensitive to distinguish whether no difference or the hypothesised mean difference of −2 was more likely. The robustness region, the range of expected effect sizes that support the same conclusion, ruled out a mean difference between groups > −8.5.

**Table 2:** Mean raw weekly consumption at baseline and 6-month follow-up.

	Comparator group (n = 2814)	Intervention group (n = 2788)	Overall (n = 5602)
Baseline, median (IQR Q1 and Q3∗)	63.50 (38.50–103.00)	63.50 (38.30–103.00)	63.50 (38.50–103.00)
6-month follow-up with BCF, median (IQR Q1 and Q3)	32.0 (16.5–67.00)	31.0 (14.59–66.00)	32.0 (15.80–66.00)
6-month follow-up with MI, median (IQR Q1 and Q3)	25.24 (9.69–48.91)	24.75 (8.25–45.76)	24.84 (8.32–46.15)

∗ IQR, Interquartile Range, Q1 and Q3, Quartile 1 and 3, BCF, Baseline carried forward, MI, Multiple imputation.

### Sensitivity analyses

Given the differences between those responding and not-responding at 6-month follow-up, the independent Data Monitoring Committee and previous research^36^ recommended that the most appropriate strategy to missing data was multiple imputation (a pre-registered sensitivity analysis, see Supplementary Table S8a for details on imputation). When imputing missing data there was a significant 2.00-unit reduction in weekly alcohol consumption at 6-month follow-up amongst those in the Drink Less group, compared with the comparator (F = 4.94, P = 0.026, Adjusted mean difference = −2.00, 95% CI = −3.76 to −0.24; Mean comparator group: 35.04 Mean in the intervention group: 33.04).

Other sensitivity analyses are reported in Appendix 6 (Supplementary Table S8a and b). In line with the analysis using multiple imputation, complete case analysis indicated a significantly lower weekly alcohol consumption at follow-up, after adjusting for baseline, among the intervention group compared with the comparator group (F = 4.78, P = 0.029, adjusted mean difference = −2.01, 95% CI = −3.81 to −0.21). No statistically significant difference was detected when missing data were imputed using last observation carried forward, but this analysis was very similar to the primary intention-to-treat analysis as among the missing 20%, 14% of consumption estimates were taken from baseline. In line with the primary analysis, there were no other significant differences on sensitivity analyses using a no-change-from baseline approach to missing data (Supplementary Table S8a).

There was a significant interaction between gender and group assignment (Supplementary Table S8b). Women in the intervention group had a significantly lower alcohol consumption than women in the comparator group (F = 5.12, P = 0.024; mean difference = −2.46, 95% CI = −4.58 to −0.33). There was a non-significant difference among men (F = 0.60, P = 0.44; mean difference = 1.08, 95% CI = −1.66 to 3.82) and those reporting ‘other’ gender (F = 0.609, P = 0.44; mean difference = 8.82, 95% CI = −13.33 to 30.98).

### Pre-registered secondary outcomes

The pre-registered analysis approach for secondary outcomes was the no-change-from-baseline approach for missing data. Using these assumptions, no significant differences were detected for the secondary outcomes (see Supplementary Table S9 in Appendix 7).

### Adverse events

≤0.1% of participants in both arms reported adverse events linked to participation in the trial (Table 3).

**Table 3:** Incidence of adverse events at 1- 3- and 6-month follow-up.

Type of Problem	1-month follow-up		3-month follow-up		6-month follow-up	
	Comparator	Intervention	Comparator	Intervention	Comparator	Intervention
Related to trial	<0.01% (3)	<0.01% (2)	<0.01% (3)	<0.01% (3)	<0.01% (1)	<0.01% (3)
Unclear link with trial	<0.01% (6)	<0.01% (9)	<0.01% (16)	<0.01% (20)	<0.01% (14)	<0.01% (14)
Unrelated to trial	<0.01% (8)	<0.01% (4)	<0.01% (9)	<0.01% (4)	<0.01% (8)	<0.01% (4)
Not a medical problem	<0.01% (3)	<0.01% (5)	<0.01% (5)	<0.01% (9)	<0.01% (1)	<0.01% (2)

### Unplanned secondary analysis

When using multiple imputation for all secondary outcomes, a weekly 2.00-unit difference was found between groups at 1-month follow-up (F = 4.10, P = 0.043, adjusted mean difference = −1.95, 95% CI = −3.85 to −0.06) and 3-month follow-up (F = 3.80, P = 0.051, adjusted mean difference = −1.78, 95% CI = −3.58 to −0.01). There were no other differences (Appendix 7: Supplementary Table S10).

An unplanned sensitivity per-protocol analysis was conducted which compared those using the app in the intervention group to all those in the comparator group, representing the best-case scenario. A statistically significant difference between groups was also found (see Supplementary Table 10). On average, those in the intervention group had a 3-unit lower consumption at 6-month follow-up compared with those in the comparator group.

## Discussion

There is evidence that Drink Less may be effective in helping increasing-and-higher-risk drinkers who are motivated to reduce their consumption to reduce their weekly alcohol consumption. Although there was not a significant difference between groups in the primary analysis, sensitivity analyses using MI showed the Drink Less app intervention group reduced their weekly alcohol consumption at 6-month follow-up by an additional two units compared with the comparator group. Given the pattern of missing data, multiple imputation was considered to be the most appropriate way to handle the missing data^36^ in this trial. This pattern of results was consistent at all time points, with an additional two unit decrease in weekly alcohol consumption at 1- and 3-month follow-ups compared with the NHS alcohol advice webpage. There was no evidence that the Drink Less app reduced the prevalence of heavy episodic drinking at 6-month follow-up. There was similarly no change in full AUDIT score after six months and no evidence that the Drink Less app impacted on any of the broader secondary outcomes in terms of; alcohol-related problems, use of healthcare services or health-related quality of life. Finally, Drink Less appears to be equally effective for increasing-and-higher-risk drinkers across all levels of baseline alcohol consumption.

The two-unit weekly reduction seen in this RCT is in line with reviews suggesting that digital interventions can have a statistically significant effect^4^ on alcohol consumption among increasing-and-higher-risk drinkers. Two reviews of digital alcohol interventions published in 2017 and 2020^4^^,^^37^ found only one other app focused on a general population had been tested in an RCT. This app resulted in no significant difference using a no-change-from-baseline approach to missing data, but detected a significant reduction of around one drink per week in a per-protocol analysis.^38^ A recent trial of an alcohol reduction app among university students in Switzerland found a reduction of one standard drink among app users (equivalent to 2.5 UK units), which is roughly equivalent to the weekly 2.00-unit reduction we see in this study.^7^

An additional two-unit-a-week decrease at six months relative to usual digital care may seem a relatively small effect. However, a weekly two-unit reduction is important because there is a dose response relationship between how much an individual drinks and their likelihood of experiencing harms.^1^ A cost-effectiveness analysis, reported separately, showed that a large-scale rollout of the Drink Less app was health improving, cost-saving and reduced health inequalities. In two roll-out scenarios, Drink Less, was estimated to save the NHS between £299 and £520 million over a 20-year time horizon.^33^

When the primary analysis was stratified by gender, there was some evidence that the app was more effective for women than for men. This may be because women tend to have higher long-term engagement with digital interventions than men^39^ and engagement can moderate mechanism of actions for intervention effectiveness.

Digital exclusion^40^ is an important factor to consider when promoting digital interventions. Some people may be unable to afford devices or data or be unable to make the most of them due to lack of knowledge. Others may not have a suitable, private environment or have access to communal IT. A report found 6% of households in the UK did not have internet access, amongst those with internet access, 8% said they were not confident in using the internet.^40^ Digital exclusion is more likely in vulnerable populations including older people, those out of work or financially vulnerable, and those who live with a physical or mental condition that limits or impairs their use of communication services. This will be an important factor to consider in any roll out of Drink Less or other digital interventions. However, with a behaviour as complex and socially engrained as alcohol consumption, it is unlikely that there is a single intervention that will help all increasing-and-higher-risk drinkers. Instead, the focus should be on providing as many effective options as is possible for people motivated to change their behaviour alongside finding effective methods for boosting motivation to reduce consumption amongst those who are not.

This study is the first remote RCT of an alcohol reduction app for the general population in the UK. Its strengths include a large sample and strict controls to reduce bias. Remote trials better represent engagement with digital interventions in the real world, without the need to travel to baseline or follow-up appointments, increasing external validity. The lack of researcher involvement in randomisation may also reduce the risk of bias and remote trials allow elements of automation, reducing the overall cost.

There are limitations of this trial. The focus is on self-reported consumption over a retrospective period. Missing data amongst 20% of the sample at 6-month follow-up is likely to have resulted in measurement and selection bias in the intention to treat analyses. Although at time of pre-registering the analysis we acted in accordance with our best knowledge, we have since become aware that assuming no-change-from-baseline has been shown to be associated with higher levels of bias than multiple imputation. Future trials should consider pre-registering primary analyses using MI approaches, particularly when data is not missing completely at random. Furthermore, an error was made on the response options for questions 1 and 2 of the AUDIT. For question 1 this led to imprecision. Rather than the response option ‘2–4 times per month’, in this study the related response option was ‘weekly’. As such it might be that more individuals drinking twice a month would have selected ‘monthly’, as opposed to ‘weekly’ in this study than would have selected ‘monthly’ relative to ‘2–4 times a month’. This could have resulted in a proportion of drinkers to have a lower AUDIT score than had the correct response options been used. On question 2 of the AUDIT, the extended options, allowing for more precision when calculating weekly consumption for heavier drinkers, were not measured until January 2021. However, this data was collected for most participants and imputed where it was missed and therefore has limited impact on the findings of this trial. Finally, though we recommended that participants scoring >20 on AUDIT at baseline sought further help, we did not assess the proportion who sought further treatment.

In terms of next steps for the Drink Less app, it would be useful to conduct implementation research examining how the Drink Less app could supplement standard healthcare in the UK. Drink Less represents part of a potential digital prevention offer which could be offered at low marginal cost to supplement face-to-face treatments. Issues directly relevant to roll-out, including process papers examining intervention engagement and mechanisms for alcohol reduction alongside short- and long-term health economic evaluations, are forthcoming. Apps require ongoing maintenance, something which cannot be administered by research grants. The app is also only currently available on Apple devices, extension to Android would be valuable.

Overall, there is evidence to suggest that the Drink Less app may be effective for reducing alcohol consumption among increasing-and-higher-risk drinkers who are motivated to reduce their consumption, compared with usual digital care.

## Contributors

MO: Methodology Equal, Investigation Equal, Data curation Equal, Validation Equal, Writing original draft Lead, Writing review & editing Equal. EB: Conceptualization Supporting, Funding acquisition Supporting, Methodology Equal, Data curation Equal, Formal analysis Lead, Writing review & editing Equal. GL: Project administration Lead, Investigation Equal, Writing review & editing Equal. LD: Project administration Lead, Investigation Equal, Writing review & editing Equal. CA: Conceptualization Supporting, Funding acquisition Supporting, Methodology Equal, Writing review & editing Equal. RB: Conceptualization Equal, Funding acquisition Supporting, Methodology Equal, Writing review & editing Equal. MF: Conceptualization Equal, Funding acquisition Supporting, Methodology Equal, Writing review & editing Equal. FG: Conceptualization Equal, Funding acquisition Supporting, Methodology Equal, Writing review & editing Equal. MH: Conceptualization Equal, Funding acquisition Supporting, Methodology Equal, Writing review & editing-Equal. EK: Conceptualization Equal, Funding acquisition Supporting, Methodology Equal, Writing review & editing Equal. SM: Conceptualization Equal, Funding acquisition Supporting, Methodology Equal, Writing review & editing Equal. MM: Conceptualization Equal, Funding acquisition Supporting, Methodology Equal, Writing review & editing Equal. EP: Conceptualization Supporting, Funding acquisition Supporting, Methodology Equal, Writing review & editing-Equal. JB: Conceptualization Equal, Funding acquisition Equal, Methodology Equal, Writing review & editing Equal. CG: Conceptualization Equal, Funding acquisition Equal, Methodology Equal, Data curation Equal, Validation Equal, Writing review & editing Equal. To confirm, both MO and CG have access to and verify the underlying study data reported in the manuscript.

## Data sharing statement

The trial was registered (ISRCTN64052601), the data analysis plan was pre-registered^22^ and updates logged with the NIHR^23^ before data were unblinded. Anonymised data, code and data dictionary are available online at OSF (https://osf.io/2j9df/).

## Declaration of interests

All authors have completed the Unified Competing Interest form (available on request from the corresponding author).

CA, MM, GL, LD, MF, EB, EP and SM declare no conflicts of interest.

MO's salary was partially funded by Medical Research Council (MR/W026430/1) and is currently a Study for the Society of Addiction funded Griffith Edwards academic fellow. MO and CG have done paid consultancy work for the behaviour change and lifestyle organization, ‘One Year No Beer’, providing fact checking for blog posts. JB has received unrestricted research funding to study smoking cessation from Pfizer and J&J, who manufacture smoking cessation medications and sits in an unremunerated role on the scientific advisory board for the SmokeFree app. FG is employed by NICE and previously by Public Health England PHE; he has no other conflicts of interest. RB is currently employed by the Office for Health Improvement and Disparities. MH is co-director of NIHR Health Protection Research Unit in Behavioural Science and Evaluation and a trustee for the Society for the Study of Addiction. EK is Director of the NIHR funded Applied Research Collaboration Northeast and North Cumbria and outside the submitted work has previously co-authored papers that analysed raw market research consumer-based data provided to Newcastle University under a direct contract with Kantar Worldpanel at no cost to Newcastle University. Kantar Worldpanel received reimbursement from AB InBev to cover the costs of the data, Kantar WordPanel having similar commercial relationships with other customers who pay to have data collected on food and non-food items available for sale in supermarkets and other retail outlets covered by the WorldPanel.

The authors declare no other support from any organisation for the submitted work; no financial relationships with any organisations that might have an interest in the submitted work in the previous three years and no other relationships or activities that could appear to have influenced the submitted work.
