# Supplementary material for: Effectiveness of a smartphone app (Drink Less) versus usual digital care for reducing alcohol consumption among increasing-and-higher-risk adult drinkers in the UK: a two-arm, parallel-group, double-blind, randomised controlled trial
Source: eClinicalMedicine. 2024 Mar 24;70:102534. doi: 10.1016/j.eclinm.2024.102534 (PMC11056393; doi:10.1016/j.eclinm.2024.102534)
Supplement: Appendix [file mmc1.pdf]

Appendices

Below we have coded the behaviour change techniques present in the Drink Less app and the NHS alcohol advice webpage. This was done in the first instance by MO, who has not undergone training in Behaviour Change Technique coding but was verified with CG who has completed Behaviour Change Technique coding training.

Appendix 1: Behaviour Change Techniques in intervention and comparator

Supplementary Table 1: Behaviour Change Techniques present in Drink Less modules

Module<br>"app name"	BCTs	Objectives/Key features	Details of module
Goal setting<br>*"Create and view goals"*	1.1 Goal setting (behaviour)	Allow users to set different weekly goals and provide information on setting appropriately specific and difficult goals.	Set weekly goals for alcohol units, alcohol-free days, calories from alcohol, spend on alcohol.
Normative Feedback<br>*"Review your drinking"*	2.7 Feedback on outcomes of behaviour<br>6.2. Social comparison	Inform users of the social drinking norm and alert them to any discrepancy with how they believe their drinking compares with normal to how it actually compares with normal.<br>Feedback on how drinking actually compares; feedback on how they think drinking compares.	Questions assessing how users think they compare with others.<br>Infographics illustrating how user's drinking actually compares with other adults and others of same gender and age.
Action Planning<br>*"Create and view action plans"*	1.4 Action planning<br>9.1 Credible source	Allow users to create implementation intentions for dealing with difficult drinking situations.	Create implementation intentions, review implementation intentions already created, gain understanding of why to

		Allow users to create/review action plans; provides examples of action plans; rationale for creating action plans.	set implementation intentions.
Self-monitoring and Feedback	1.5 Review behaviour goals<br>1.6 Discrepancy between current behaviour and goal<br>2.2 Feedback on behaviour<br>2.3 Self-monitoring of behaviour<br>2.4 Self-monitoring of outcomes of behaviour<br>2.7 Feedback on outcomes of behaviour<br>5.2 Salience of consequences<br>5.6 Information about emotional consequences<br>10.3 Non-specific reward<br>10.4 Social reward<br>10.9 Self-reward	Facilitate easy and on-going recording of alcohol consumption; provide feedback on consumption, consequences of consumption and progress against goals.<br>Allow users to monitor their consumption; provides feedback on consumption; feedback on consequences of consumption (mood, productivity, sleep)	Ability to record drinks, graph showing units consumed calories consumed, amount spent on alcohol. Record mood, productivity, clarity, sleep quality, graph illustrating how they differ on mornings after heavy drinking compared to mornings after light/no drinking. Feedback on progress towards goals: cumulatively as the week progresses, on the past week and on all previous weeks.
Information about antecedents	4.2. Information about Antecedents	Prompts users to identify the situations and events, or feelings that occur before drinking.	Users can select from a list of drinking cues and add their own to identify the situations and events, or feelings that occur before drinking.

"Your Drinking Cues"			Users are then prompted to make a plan for how to deal with these drinking cues and signposts the 'Action Planning' module.
Behaviour substitution<br>*"Drinking Alternatives"*	8.2. Behaviour substitution	Prompts users to make an alternative plan.	Prompts users to make an alternative plan within the drinking calendar on the app (e.g., go for a walk, watch a film, have a soft drink). Users can choose from a list of options or write their own, and set a reminder if they wish to.
Insights	1.5. Review behaviour goal(s)<br>2.2. Feedback on behaviour	To provide an overview of users' drinking habits including a more detailed summary of weekly and/or monthly feedback.	Three sections of the module "My Last Week", "My Average Week", "Lifetime Totals".<br>My Last Week and Lifetime Totals showing the total number of units, money spent, calories consumed, and alcohol-free days as well as a summary of their goals. My Average Week shows their average week in terms of units, calories, cost, and alcohol free days over different user-selected time periods: one month, three months, six months, one year, lifetime, with a graphical representation

			of their weekly units and alcohol free days.
Cognitive Bias Re-training<br>*"Game"*	No individual BCTs are directly relatable to this intervention module. In combination, the following are of relevance:<br>7.8 Associative learning<br>8.1 Behavioural practice/rehearsal<br>8.3 Habit formation	Use a form of cognitive bias modification to strengthen cognitive control over the automatic biases to approach alcohol that predict alcohol use that exist amongst the users through an engaging game.	Game with all alcohol related pictures associated with "avoid" and all soft drink pictures associated with "approach".<br>Additional section of text on why and how this sort of game is believed to work.

Supplementary Table 2: Behaviour Change Techniques present in NHS Alcohol Advice webpage summary

BCTs	Description
1.1 Goal setting (behaviour)	Encourages users to have several alcohol-free days each week.
1.3 Goal setting (outcome)	Encourages users to set consumption and financial goals.
1.4 Action planning	Suggests ordering smaller drinks (a bottle of beer rather than a pint) or switching to a lower ABV when drinking.

3.1 Social support (unspecified)	Suggests telling family and friends to receive support in achieving goals.
5.1 Information about health consequences	Outlines benefits to heart, immune system, memory, weight management and sleep.
5.3 Information about social and environmental consequences	Suggests drinking can result in aggressive or irrational behaviour.
5.6 Information about emotional consequences	Discusses link between drinking and depression and benefits to cutting down.
9.1 Credible Source	NHS generally agreed on as credible source of health advice.

Appendix 2: Additional Procedural detail

Follow-up

For the 1- and 3-month follow-up surveys, participants were sent up to four automated emails from days 0, 5, 9 and 11. From 01/15/2022, participants also received a text message reminder at the same time as the second (day 5) and fourth (day 11) email. At six months, as well as three emails (days 0, 5 and 9) and (from 01/15/2022) two text messages (days 5 and 9), participants who did not complete the web-based follow-up assessment were sequentially offered opportunities to do so via phone (called twice from days 10-17), mailed survey (from day 18) and mailed postcard (from day 30). Participants were compensated with gift vouchers of up to £36 for completing the three surveys: £6 for the 1- and 3-month follow-up; £12 for the 6-month follow-up with an additional £12 if it was completed within 24 hours.

Data Cleaning

There were four data sheets linked by a pseudonymised participant ID. One containing the baseline data, plus three from the 1-, 3- and 6-month follow-ups. These four sheets were merged using pandas. There were problems throughout the study with bot responses (automated responses), manual participant deception (participants signing up multiple times with false information) and duplicates (participants signing up multiple times with duplicate information), presumably motivated by the financial incentives in this study. The process for identifying and removing participants providing false or duplicate information is described in a separate paper. Those identified as a bot, manual deception, duplicate (second responses) or who withdrew were removed from the file. All personally identifiable information was removed before the file was shared with the trial statistician.

Appendix 3: Coding for AUDIT questions 1 and 2 and weekly unit derivation.

An error was made on questions 1 and 2 of the AUDIT questionnaire. For details on non-standard response options for questions 1 and 2 as measured in this study see Supplementary Tables 1 and 2. AUDIT questions 3-10 are as standard.

Due to this error, extended responses were not collected until the 01/15/2021 for participants selecting '10 or more units' to question 2 of the AUDIT. This equates to 656 participants (12% of the total expected) at baseline, 186 (3%) at 1-month, 79 (1%) at 3-month and 1 (<.1%) at 6-month follow-up. These data were imputed, following recommendations 20 imputed datasets were used and combined using Rubin's rules[26]. To resolve collinearity, ethnicity was dichotomised into White versus Other, education was dichotomised into 16+ qualifications and no 16+ qualifications, and the *prefer not to say* category for gender was coded as missing. We assessed the bias of our imputation method by comparing the estimates obtained after imputation with the 'true' parameter value for a set of participants who did not have missing data.

Supplementary Table 3: Overview of original AUDIT-C questions, extended AUDIT-C questions and those used in the iDEAS trial and how they will be scored for AUDIT q1 'How often do you have a drink containing alcohol?'

Original AUDIT-C q1	Extended AUDIT-C q1	AUDIT-C q1 used in iDEAS trial	Scoring for full adapted AUDIT score
Never	Never	Never	0
Monthly or less	Monthly or less	Less than monthly	1
		Monthly	1
2-4 times per month	2-4 times per month	Weekly	2
2-3 times per week	2-3 times per week	2-3 times a week	3
4+ times per week	4-5 times per week	4-6 times a week	4

	6+ times per week	Daily	4

Supplementary Table 4: Overview of original AUDIT-C questions, extended AUDIT-C questions and those used in the iDEAS trial and how they will be scored for AUDIT q2 'How many units of alcohol do you drink on a typical day when you are drinking?'

Original AUDIT-C q2	Extended AUDIT-C q2	AUDIT-C q2 used in iDEAS trial up until 15/1/21	AUDIT-C q2 used in iDEAS trial from 15/1/21 onwards	Scoring for full adapted AUDIT score
0-2	0-2	1	1	0
		2	2	0
3-4	3-4	3	3	1
		4	4	1
5-6	5-6	5-6	5-6	2
7-9	7-9	7-9	7-9	3
10+	10-12	10+	10-12	4
	13-15		13-15	4
	16+		16+	4

Supplementary Table 5: Weekly unit derivation from AUDIT-C with non-standard response options as measured in this trial.

Frequency (AUDIT-C q1)		Quantity (AUDIT-C q2)		Heavy Episodic Drinking (AUDIT-C q3)[d]	
Response option	Score	Response option	Score	Response option	Score
Never	0[a]	1 units	1	Never	0
Less than monthly	0.0729[b]	2 units	2	Less than monthly	0.8019
Monthly	0.25	3 units	3	Monthly	2.75
Weekly	1	4 units	4	Weekly	11
2-3 times a week	2.5	5-6 units	5.5	Daily or almost daily	33
4-6 times a week	5	7-9 units	8		
Daily	7	10-12 units[e]	11[c]		
		13-15 units	14		
		16+	21		

[a] if AUDIT-C q1 = Never (0), then weekly alcohol consumption =0 regardless of other responses

[b] midpoint between yearly (after which participants should respond never) and once every two months - so average of (((1/12)/4)+(0.5/4))/2 = 0.0729

[c] Extended response options (10+) will be imputed for participants responding to baseline survey and follow ups before 01/15/2021 due to an error.

[d] AUDIT-C q3: "How often you have six or more drinks on one occasion?" where a drink = 1 unit. Because it's "six or more" we add 5 to be consistent with treatment of "or more" in the quantity question and use the frequencies as above (except for daily or almost daily, which is calculated as the average difference between daily and weekly)
Never = 0
Less than monthly = 0.0729 x (6+5) = 0.8019
Monthly = 0.25 x (6+5) = 2.75
Weekly = 1 x (6+5) = 11
Daily or almost daily = (7+3.5)/2 x (6+5) = 57.75

Appendix 4: Additional detail on COVID-19 and adverse events measures

Supplementary table 6: COVID-19 measures

Question	Response Options
Do you currently feel like COVID-19 is affecting your alcohol consumption and how you feel about drinking alcohol?	Yes/No<br><br>Participants responding “yes” will be asked to answer five follow up questions
Is COVID-19 and its associated effects (e.g. financial, social or health) currently affecting how worried you feel about your alcohol consumption?	more worried<br>no change<br>less worried
Is COVID-19 and its associated effects currently affecting your motivation to reduce your alcohol consumption?	more motivated<br>no change<br>less motivated’
Is COVID-19 and its associated effects affecting how frequently you consume alcohol?	consume alcohol more frequently<br>no change<br>consume alcohol less frequently

Is COVID-19 and its associated effects currently affecting how many units of alcohol you generally consume when you do drink?	generally drink more units<br>no change<br>generally drink less units
Is COVID-19 and its associated effects currently affecting how often you consume 6 or more units of alcohol on a single occasion?"	more likely to consume 6 or more units on a single occasion<br>no change<br>less likely to consume 6 or more units on a single occasion

Safety and Adverse Events

Participants were asked to report any unexpected consequences, adverse events or other harms from participating in the study (in an open-ended question at the 1-, 3- and 6-month follow-up). Free text responses underwent content analysis and were categorised as to whether they were;

1) A medical problem related to participation in the trial or cutting down where issue is linked to cutting down (e.g. 'withdrawal') or participants explicitly link medical issue to study or to cutting down, e.g. 'My mental health has deteriorated thinking about alcohol and feeling a failure for drinking too much.'

2) A medical problem with an unclear link to participation in the trial or cutting down. Participants note a medical group but did not explicitly link their experience of these to cutting down or the study e.g. 'bowel problems'.

3) Medical Problem unrelated to participation in the trial or cutting down where participants noted a medical issue unrelated to study or cutting down. This could be related to heavy drinking such as accidents when drunk (e.g. 'I was drunk so went to bed and knocked myself out on the door skirting breaking my nose in 3 places and broken cheekbone, lost lots of blood and rushed to A & E') or advice from a doctor suggesting they cut down (e.g. 'Because I drink too much, for my health, the doctor recommended that I drink less').

4) Not a medical problem. This is where participants indicated they hadn't experienced any medical problems (e.g. 'sorry this was a mistake', or 'none'). This could also be where something is listed which is not a medical problem (e.g. 'Made me look at myself and decided to change which is gradually working').

Appendix 5: COVID-19 Measures

Supplementary Table 7: COVID-19 measures at baseline by group and overall

Variable	Overall (n=5,602)	Comparator group (n=2,814)	Intervention group (n=2,788)	*P*
COVID affecting alcohol consumption				
Yes	66.46 (3723)	65.17 (1834)	67.75 (1889)	0.044
No	33.54 (1879)	34.83 (980)	32.25 (899)	
Impact of COVID on concerns about alcohol consumption[a]				
More worried	71.69 (2669)	71.70 (1354)	71.68 (1354)	0.22
No change	25.17 (937)	25.03 (459)	25.30 (478)	
Less worried	3.14 (117)	3.27 (60)	3.02 (57)	
Impact of COVID on drinking frequency[a]				
More frequently	82.41 (3068)	82.55 (1514)	82.27 (1554)	0.56
No change	12.25 (456)	12.49 (229)	12.02 (227)	
Less frequently	5.35 (199)	4.96 (91)	5.72 (108)	
Impact of COVID on unit consumption[a]				
Drink more units	73.78 (2747)	73.94 (1356)	73.64 (1391)	0.80
No change	20.90 (778)	20.56 (377)	21.23 (401)	
Drink less units	5.32 (198)	5.51 (101)	5.12 (97)	
Impact of COVID on drinking 6+ units on one occasion[a]				
More likely	70.64 (2630)	70.99 (1302)	70.30 (1328)	0.86
No change	24.55 (914)	24.15 (443)	24.93 (471)	
Less likely	4.81 (179)	4.85 (89)	4.76 (90)	
Impact of COVID on motivation to reduce drinking[a]				
Less motivated	45.50 (1694)	45.42 (833)	45.58 (861)	0.91
No change	21.54 (802)	21.32 (391)	21.76 (411)	
More motivated	32.96 (1227)	33.26 (610)	32.66 (617)	

Notes: reported %(n). P values based on chi-squared test. [a]Only asked if participants reported that covid was affecting their alcohol consumption.

Appendix 6: Pre-registered Sensitivity Analyses

Some of the pre-registered sensitivity analyses took a different approach to missing data than the more conservative approach of assuming no-change-from-baseline. In line with the analysis using multiple imputation, complete case analysis indicated a significantly lower weekly alcohol consumption at follow-up, after adjusting for baseline, among the intervention group compared with the comparator group. No statistically significant difference was detected when missing data were imputed using last observation carried forward. However, due to lower rates of 1- and 3-month follow-up than at 6-month follow-up, this analysis was very similar to the primary intention-to-treat analysis as among the missing 20%, 14% of consumption estimates were taken from baseline.

Other pre-registered sensitivity analyses focused on examining a variation on the outcome variable or the analysed sample, but missing data were treated in the same way as the primary analysis (i.e. no change from baseline). When using a change score between baseline and 6-month follow-up as the outcome, no statistically significant difference was found between groups (see Table 3a). The per-protocol analysis was based on those in the intervention group having self-reported use of the Drink Less app at one and six months. It was also based on those in the comparator group having self-reported using the NHS alcohol advice webpage at one or six months but excluding those having downloaded the Drink Less app (an indication of contamination). No difference was found between groups in weekly alcohol consumption. In the instrumental variable analysis, correct app usage (using Drink Less in the intervention group and using the NHS alcohol advice webpage in the comparator group but not using Drink Less in the comparator group) was regressed onto group randomisation, yielding the predicted values of usage. These represent usage as a function of being randomised or not, and thus aims to exclude any inherent selection bias. Next, weekly alcohol consumption was regressed onto the predicted usage and adjusted for baseline values. The goal to provide an unbiased estimate of the link between usage, as influenced by randomisation, and consumption. The first regression model predicted that all those randomised to the intervention group had used the app and all those randomised to the comparator group had not used the app. The analysis therefore closely mirrors the primary analysis and finds no statistically significant difference between groups. This is due to the low levels of contamination in the comparator group compared to Drink Less usage in the intervention group. Finally, there was no evidence of interactions between group allocation and baseline drinking on the primary outcome variable, suggesting the *Drink Less* app was not differentially effective for lighter or heavier drinkers.

Supplementary Table 8a: Results of the sensitivity analyses

Analysis	*Statistics*[a]
Change between baseline and 6-month follow-up in weekly alcohol consumption	P=0.18<br>Mean difference: -1.30 (95%CI -3.20 to 0.60)
Complete case analysis with weekly alcohol consumption at 6-month follow-up adjusted for baseline consumption	P=0.029<br>Adjusted mean difference: -2.01 (95%CI -3.81 to -0.21)
Weekly alcohol consumption 6-month follow-up adjusted for baseline consumption with last observation carried forward[d]	P=0.22<br>Adjusted mean difference: -1.14 (95%CI -2.82 to 0.55)
Per-protocol analysis of weekly alcohol consumption 6-month follow-up adjusted for baseline consumption[b]	P=0.42<br>Adjusted mean difference: -0.71 (95%CI -3.39 to 0.98)
Instrumental variable analysis using group randomisation as the instrument and adjustment for baseline consumption in stage 2	P=0.25<br>Beta=-0.98 (95%CI -2.67 to 0.71)
Interactions between group allocation and baseline drinking (using natural cubic splines) on alcohol consumption at 6-month follow-up[c]	
*Group*Weekly alcohol consumption [0,54.6]*	P=0.24, Beta= -7.43 (95%CI -19.94 to 5.08)
*Group*Weekly alcohol consumption [54.6,180]*	P=0.24, Beta= -4.69 (95%CI -12.57 to 3.09)
Counterfactual Analysis excluding those who reported not having IOS after randomisation (n=21) and the first responses of duplicate respondents (n=43)	P=0.18<br>Adjusted mean difference of -1.16 (95%CI -2.85 to 0.50)

Note:[a] Beta coefficient values from linear regression (using the glm framework; assumptions of normality, homogeneity of variance of residuals and linearity for quantitative predictors were tested using histograms, boxplot, Bartlett's test and plotting predictors and were met).[b] Analysis based on n=1,782 in the intervention group and n=1,554 in the comparator group; [c]Number of knots derived through leave-one-out cross-validation, three knots were placed at equally spaced quantiles of data. [d] based on 120 having missing data at 6 months being imputed from 3 months and a further 160 having missing data at 6 months being imputed from 1 month.

Supplementary Table 8b: Results of the sensitivity analyses

Analysis	*Statistics (interaction term)*
Interaction between participant characteristics and group allocation (intention to treat)	
*Group*Age*	Beta=-0.01 (95%CI -0.15 to 0.12), P=0.83.
*Group*gender (Ref Female)*	
Other	Beta=11.20 (95%CI -13.65 to 36.07), P=0.38
Male	Beta=3.52 (95%CI 0.10 to 6.93), P=0.044
*Group*ethnicity (Ref White)*	
Other	Beta=-2.45 (95%CI -10.11 to 5.19), P=0.53
*Group*education (Ref No post-16 qualfications)*	
Post-16 qualifications	Beta=0.40 (95%CI -8.34 to 9.15), P=0.93
*Group*AUDIT score*	Beta=2.24 (95%CI -2.44 to 6.86), P=0.35
*Group*occupation (Ref High man, admin or*	
professional)	Beta=2.21 (95%CI 0-2.44 to 6.86), P=0.35
Intermediate managerial, administrative or	Beta=0.40 (95%CI -8.21 to 9.01), P=0.93
professional	Beta=-2.28 (95%CI -9.57 to 5.01), P=0.54
Semi and unskilled manual workers	Beta=-1.53 (595%CI -11.00 to 7.95), P=0.75
Skilled manual workers	Beta=-0.31 (95%CI -5.42 to 4.80), P=0.91
State pensioners	Beta=-1.30 (95%CI -7.56 to 4.95), P=0.68
Supervisory, clerical and junior managerial, administrative	
Unemployed	Beta=-2.59 (95%CI -6.47 to 1.30), P=0.19
*Group*income (Ref More than £26k)*	
Less than £26k	Beta=0.41 (95% -3.15 to 3.98), P=0.82
*Group*covid affecting alcohol consumption (Ref Yes)*	
No	

Appendix 7: Secondary outcomes using pre-registered no-change-from-baseline and multiple imputation approaches to missing data

Supplementary Table 9: Results of the secondary outcomes using pre-registered no-change-from-baseline approach to missing data

Analysis	*Statistics*
Weekly alcohol consumption at 1-month follow-up adjusted for baseline consumption	P=0.73<br>Adjusted mean difference: -0.28 (95%CI -1.79 to 1.24)
Weekly alcohol consumption at 3-month follow-up adjusted for baseline consumption	P=0.78<br>Adjusted mean difference: 0.23 (95%CI -1.36 to 1.82)
Heavy episodic alcohol use (measured using AUDIT question 3) at 6-month follow-up	P=0.13<br>Mean difference: -0.05 (95%CI -0.12 to 0.01)
Full adapted AUDIT score at 6-month follow-up	P=0.47<br>Mean difference: -0.05 (95%CI -0.12 to 0.01)
Alcohol-related problems or consequences and alcohol-related injury (Alcohol Short Index of Problems) at 6-month follow-up	P=0.99<br>Mean difference: -0.16 (95%CI -0.59 to 0.27)
Use of healthcare services (Service Use Questionnaire) at 6-month follow-up	P=0.11[a]<br>Mean difference: 0.32 (95%CI -0.06 to 0.71)
Health-related quality of life (EQ-5D-5L) at 6-month follow-up	P=0.75[b]<br>Mean difference: <0.01 (95%CI -0.15 to 0.15)<br>Median: 7 intervention group and 7 comparator group.
Interactions between group allocation and baseline drinking (using natural cubic splines) on alcohol consumption at 3-month follow-up	
*Group*Weekly alcohol consumption [0,51]*	P=0.25, Beta=-7.93 (95%CI -21.49 to 5.62)
*Group*Weekly alcohol consumption [51,180]*	P=0.54,Beta= 4.90 (95%CI -10.60 to 20.41)
Interactions between group allocation and baseline drinking (using natural cubic splines) on alcohol consumption at 1-month follow-up	
*Group*Weekly alcohol consumption [0,180]*	P=0.96, Beta=-0.28 (95%CI -10.15 to 9.58)

Note: [a]Welch's ANOVA with unequal variances; [b]Kruskal-Wallis is run as the non-assumption alternative to the One-way ANOVA with violation of the assumption of normality.

Supplementary Table 10: Results of the secondary outcomes using multiple imputation

Analysis	*Statistics*
Per-protocol analysis (only intervention group[c]) of weekly alcohol consumption 6-month follow-up adjusted for baseline consumption	P=<0.0001<br>Adjusted mean difference: -2.94 (95%CI -4.71 to -1.18)
Weekly alcohol consumption at 1-month follow-up adjusted for baseline consumption	P=0.043<br>Adjusted mean difference: -1.95 (95%CI -3.85 to -0.06)
Weekly alcohol consumption at 3-month follow-up adjusted for baseline consumption	P=0.051<br>Adjusted mean difference: -1.78 (95%CI -3.58 to -0.01)
Heavy episodic alcohol use (measured using AUDIT question 3) at 6-month follow-up	P=0.24<br>Mean difference: -0.04 (95%CI -0.10 to 0.03)
Full adapted AUDIT score at 6-month follow-up	P=0.32<br>Mean difference: -0.21 (95%CI -0.63 to 0.20)
Alcohol-related problems or consequences and alcohol-related injury (Alcohol Short Index of Problems) at 6-month follow-up	P=0.69<br>Mean difference: -0.08 (95%CI -0.56 to 0.39)
Use of healthcare services (Service Use Questionnaire) at 6-month follow-up	P=0.22[a]<br>Mean difference: 0.22 (95%CI -0.13 to 0.56)
Health-related quality of life (EQ-5D-5L) at 6-month follow-up	P=0.53[b]<br>Mean difference: -0.07 (95%CI -0.25 to 0.10)<br>Median: 6 intervention group and 6 comparator group.
Interactions between group allocation and baseline drinking (using natural cubic splines) on alcohol consumption at 3-month follow-up	
*Group*Weekly alcohol consumption [0,51]*	P=0.23, Beta=-7.98 (95%CI -21.02 to 5.06)
*Group*Weekly alcohol consumption [51,180]*	P=0.71, Beta= 3.62 (95%CI -5.72 to 8.46)
Interactions between group allocation and baseline drinking (using natural cubic splines) on alcohol consumption at 1-month follow-up<br>*Group*Weekly alcohol consumption [0,180]*	P=0.82, Beta=2.66 (95%CI -20.63 to 25.94)

Note: [a]Welch's ANOVA with unequal variances; [b]Kruskal-Wallis is run as the non-assumption alternative to the One-way ANOVA with violation of the assumption of normality; [c]self-report use and downloaded; sociodemographic differences predicted missingness on SIPS, healthcare service use and quality of life. Indicating at least Missing at Random and perhaps Missing Not at Random
